# Heterogenesis

**Published:** 1902-07-26

**Authors:** 


### The Hospital

A Journal of

The Medical Sciences and Hospital Administration.

Vol. XXXII.—No. 826. Edited by Sir Henry Burdett, K.C.B., and Solomon C. Smith, M.D., M.R.C.P. [July 26, 1902.

### "Heterogenesis."

The recent publication by Dr. Charlton Bastian of the second series of his "Studies in Heterogenesis," with an appended note concerning the refusal of the Royal Society, of which he is a Fellow, to give a hearing to a communication on the subject, together with the publication of the first results of the investigation initiated by Dr. Latham into the therapeutical properties of *Lacnanthes*, may serve to raise very interesting and important questions concerning the extent to which it may be incumbent upon philosophers to take note of opinions or assertions, in relation to the subject matters of their studies, which appear to be subversive of doctrines almost universally held to be true. Dr. Charlton Bastian defines heterogenesis as "the production from the substance of organisms or their germs of alien forms of life," and he gives, as one of the most important of his illustrations of the alleged variation, "the transformation, in the course of three or four days, of the entire contents of the egg of Hydatina senta into a large ciliated infusorium belonging to the genus Otostoma." Modern biology does not admit the possibility of the event which Dr. Bastian describes, and the successive steps of which he illustrates by a series of well-executed micro-photographs, and its representatives in the Royal and other learned societies practically refuse to discuss the question, or to make any attempt to discover and point out the sources of error by which, in their judgment, the recorded experiments must have been invalidated. It may undoubtedly be alleged, on the part of the biologists, that Dr. Bastian has held and advocated beliefs concerning spontaneous generation which were investigated by the late Professor Tyndall and others, and were shown to be devoid of foundation; and that consequently he may be regarded as an observer by no means exempt from human liability to error, whose conclusions do not possess sufficient weight to justify departures from useful work in other directions for the mere purpose of showing in what manner he has been misled. On the other hand, when Mr. Alabone, of whom nothing is known but that he was once a member of the Royal College of Surgeons, who was deprived of his membership for reasons held to be sufficient by the Council, and who has since carried on the trade of an unqualified advertising practitioner of medicine, alleged that a certain plant named *Lacnanthes tinctoria* was calculated to exert a curative influence over pulmonary phthisis, his assertion was held, not only by uninstructed public opinion, but even by a distinguished Cambridge professor, to justify and require an examination of the grounds upon which it was supposed to rest. We presume that no physician would regard the truth of the assertion made by Mr. Alabone to be probable, or even possible, for the apparently sufficient reason that the bacillus of tubercle does not appear to be susceptible of injury from the presence, in the blood or tissues, of minute quantities of any agent which is not calculated to be destructive to the host itself. We must apologise to Dr. Bastian for mentioning his name and work in the same paragraph with the assertions of Mr. Alabone, but the gulf by which the two men are divided is, nevertheless, one which offers no place at which a line of demarcation can be drawn. If Dr. Latham was right in so far yielding to popular outcry as to take part in an inquiry into the properties of *Lacnanthes*, the Royal Society was clearly wrong in placing difficulties in the way of a complete discussion of the alleged heterogenesis, although it by no means follows that the converse of this proposition would hold good. But the *primâ facie* evidence against Dr. Bastian's views, as it would present itself to the mind of a biologist, cannot be so strong as that against the assertions of Mr. Alabone, as it would present itself to the mind of a physician; even leaving out of account, in the latter case, the total absence of any record of scientific work, or of any evidence of scientific accuracy, on the part of the assertor. Taken together, the two questions serve to illustrate the extreme difficulty which may arise in determining whether certain specific allegations should be made the subject of inquiry independently of their authors, and independently of the reasonable claim which may be made upon those who speak to prove their case. It is fairly certain that the biologists who refuse a hearing to Dr. Bastian would have refused a hearing to Pasteur, and that in doing so, if they could have pointed to any erroneous conclusion which he had based upon his earlier work, they would have regarded it as materially strengthening the position they had assumed. Yet the discovery of apparent exceptions, in the history of science generally, has often been a step towards the discovery of laws by which the apparent exceptions were embraced; and although at present, Dr.

Bastian is crying in the wilderness, no one can say to what extent, if any, the appearances which he has described and depicted may not lead the way to better knowledge of the conditions of life among the minute organisms which we have come to regard as holding an ancestral relation to others which are higher in the scale. If, on the other hand, the channel through which the germs of the heterogenetic growths found access to his materials can be pointed out, the hypotheses which he has founded upon their presence will die harmlessly.

